# Auditory Stimulation Dishabituates Olfactory Responses via Noradrenergic Cortical Modulation

**DOI:** 10.1155/2009/754014

**Published:** 2009-04-01

**Authors:** Jonathan J. Smith, Kiseko Shionoya, Regina M. Sullivan, Donald A. Wilson

**Affiliations:** ^1^Department of Zoology, University of Oklahoma, Norman, OK 73019, USA; ^2^Emotional Brain Institute, Nathan Kline Institute for Psychiatric Research, 140 Old Orangeburg Road, Orangeburg, NY 10962, USA; ^3^Department of Child and Adolescent Psychiatry, New York University School of Medicine, 577 First Avenue, New York, NY 10016, USA

## Abstract

Dishabituation is a return of a habituated response if context or contingency changes. In the mammalian olfactory system, metabotropic glutamate receptor mediated synaptic depression of cortical afferents underlies short-term habituation to odors. It was hypothesized that a known antagonistic interaction between these receptors and norepinephrine ß-receptors provides a mechanism for dishabituation. The results demonstrate that a 108 dB siren induces a two-fold increase in norepinephrine content in the piriform cortex. The same auditory stimulus induces dishabituation of odor-evoked heart rate orienting bradycardia responses in awake rats. Finally, blockade of piriform cortical norepinephrine ß-receptors with bilateral intracortical infusions of propranolol (100 *μ*M) disrupts auditory-induced dishabituation of odor-evoked bradycardia responses. These results provide a cortical mechanism for a return of habituated sensory responses following a cross-modal alerting stimulus.

## 1. Introduction

Dishabituation is a rapid recovery from habituation, often (though not necessarily) following an intense stimulus potentially in a different modality from that of the habituated stimulus [1]. Dishabituation allows for a return of responsiveness to filtered stimuli if context or contingency changes and thus can be highly adaptive in dynamic environments. Though habituation and dishabituation are relatively “simple” forms of memory, disruptions in these basic processes are associated with and/or could lead to higher-order cognitive, emotional, or information processing impairments [2–4].

In invertebrates, synaptic mechanisms of dishabituation include both a reversal of habituation, and in some situations, an additional sensitization of the response [5, 6]. Invertebrate dishabituation is dependent on modulatory inputs such as serotonin to the sensorimotor circuit that reverse or counteract the synaptic changes underlying habituation [5]. For example, habituation in *Aplysia* is mediated by a decrease in presynaptic neurotransmitter release from the sensory neuron onto motor neurons [7, 8]. Serotonin can reverse this depression and in nondepressed synapses induce facilitation of neurotransmitter release [9–11].

In the mammalian olfactory system, short-term habituation to odors is mediated by a metabotropic glutamate receptor (mGluR)-dependent depression of cortical afferent synapses. Both odor stimulation in vivo [12] and patterned electrical stimulation of cortical afferents in vitro [13] induce depression of the glutamatergic afferent synaptic input to piriform cortex pyramidal cells. Blockade of presynaptic mGluR group III receptors within the piriform cortex prevents this synaptic depression in vitro [13], impairs cortical adaptation to odors [13], and reduces behavioral odor habituation [14–16]. Infusion of the mGluR group III agonist AP4 into the piriform cortex induces afferent synaptic depression [14] and depresses odor-evoked reflexes (Wilson, unpublished observations). Together, these results suggest a necessary and sufficient role of piriform cortical mGluR group III receptors in short-term odor habituation.

Interestingly, Cai et al. have demonstrated that mGluR group III receptor-mediated second messenger cascades are modulated by noradrenergic ß-receptors [17]. Activation of noradrenergic ß-receptors can disrupt the effects of mGluR group III receptors. In fact, the noradrenergic ß-receptor agonist isoproterenol can block synaptic depression at in vitro piriform cortical afferents [13]. The piriform cortex, and olfactory system in general, receives a strong noradrenergic input from the locus coeruleus [18].

These results lead to the hypothesis that events which activate the locus coeruleus (e.g., novel, unexpected, or intense stimuli) could block piriform cortical afferent synaptic depression, leading to a reinstatement of odor-evoked responses dishabituation. The present experiments test this hypothesis by demonstrating that an auditory stimulus can enhance NE release in the piriform cortex and induce dishabituation of odor-evoked reflexes. Finally, the dishabituation can be blocked with infusions of a noradrenergic ß-receptor antagonist into the piriform cortex.

## 2. Materials and Methods

Adult male Long-Evans hooded rats were used as subjects. Animals were housed on a 12 : 12 light-dark cycle, with testing occurring during the light portion. Food and water were available ad lib, and housing, care, and experimental protocols conformed to NIH and University of Oklahoma IACUC standards.

Electrocardiograms were recorded with subcutaneous telemetry devices (Data Sciences, Intl.). Animals were anesthetized with isoflurane and the telemetry device was implanted under the dorsal skin near the shoulders, with electrode leads passed subcutaneously to ventral muscles at anterior and posterior locations providing strong electrocardiogram signals. Wounds were closed with sutures and animals allowed to recover approximately one week before testing. For analysis and manipulation of piriform cortical norepinephrine, in some animals guide cannulas (plastics one, 26 gauge) were also implanted into the anterior piriform cortex (unilateral for microdialysis, bilateral for drug infusions; 1.0 mm anterior to Bregma).

For testing of habituation/dishabituation, animals were placed in a darkened test chamber (either a 16 cm × 22 cm × 15 cm Plexiglas box or a 20 cm diameter × 20 cm high glass beaker. No difference was noted between results in the two chambers). The animals were allowed to acclimate for 10 minutes. Following acclimation, odor pulses were delivered (4 pulses, 2-second duration, 60-second interstimulus interval (ISI)) to determine mean baseline response magnitude (average of the response to the 4 spaced stimuli). Odorants were either isoamyl acetate or eugenol and were delivered by passing clean air over an odorant saturated (250–400 *μ*L) Kimwipe through Teflon tubing into the test chamber. The odor pulses were then continued at 20-second interstimulus intervals for 100 repetitions [19] to induce habituation (uninfused rats) or 60 trials (infused rats). The reduced number of habituation trials in the infused rats was sufficient to induce habituation (see Figure 2) but was short enough to accommodate the time required for the 20-minute drug infusions. The extent of habituation was then monitored by 4 additional odor pulses with a 60-second ISI, with the mean response magnitude to the four stimuli serving as the habituated response magnitude for each animal. Finally, experimental rats (noninfused, aCSF and propranolol infused) then received 4 final odor pulses (60-second ISI) that were each preceded by a 1–2-second auditory stimulus (siren, 108 dB, 3 kHz, RadioShack). Control animals received the same final set of odors, but no auditory stimulus. In two animals, one of the postacoustic responses was dropped due to large artifacts in the recording, thus, their mean responses were based on three stimuli rather than four. The mean response to these four stimuli was used as the measure of postacoustic dishabituation in each animal. Odor-evoked heart rate orienting response magnitude was calculated as the maximal change in instantaneous beats/minute within 10 seconds of the odor stimulus. Measures for each time point were the average of the 4 evoked responses (baseline, posthabituation, postacoustic).

During pharmacological manipulations, 5 minutes prior to behavioral testing onset (25 minutes prior to dishabituation induction), bilateral infusions were begun (0.15 *μ*L/min, 20 minutes). Five minutes after infusion onset, the 20-minute habituation protocol was begun which allowed the full 3 *μ*L infusion to be completed at least 5 minutes prior to the dishabituation induction. Drugs were infused with a syringe pump through an internal cannula (33 gauge) placed in the guide cannula. Animals were infused with either artificial cerebrospinal fluid (124 mM NaCl, 5 mM KCl, 1.24 mM KH_2_PO_4_, 2.4 mM CaCl_2_, 1.3 mM MgSO_4_, 26 mM NaHCO_3_ and 10 mM glucose) or 100 *μ*M propranolol in artificial cerebrospinal fluid. Each animal was tested with both perfusates in a counterbalanced order, with at least 4 days between consecutive tests. Following data collection, rats implanted with bilateral cannulas were transcardially perfused with saline and 10% formalin. Brains were sectioned coronally at 40 *μ*m and cannula tips localized in cresyl violet stained sections.

For microdialysis/HPLC analysis of piriform cortex NE content, on the day of testing a microdialysis probe (EICOM corp., Kyoto, Japan; A-I-8-02, 8 mm long, 2 mm membrane, 220 *μ*m diameter) was placed through the guide cannula with the tip in the left anterior piriform cortex. Microdialysis probes were inserted into the guide tube of the animal 60 minutes before data collection. During microdialysis experiments, rats were placed in a 27 cm diameter acrylic circular cage that was able to move freely with rotary bearings (EICOM corp., Kyoto, Japan), or in a 21 cm × 28 cm foot shock chamber. Microdialysis probes were continually perfused with an artificial cerebrospinal fluid (aCSF) containing 147 mM NaCl, 2.7 mM KCl, 1.2 mM CaCl_2_, and 0.85 mM MgCl_2_ in deionized water at a flow rate of 2.0 *μ*L/min using a microinjection pump (EICOM, ESP-64). Dialysates were collected and held at 4°C automatically in 10 minutes intervals into plastic microvials preloaded with 2 *μ*L of 12.5 mM perchloric acid/250 *μ*M EDTA using refrigerated fraction collector (EICOM, EFC-82). Samples (20 *μ*L) were collected during each 10 minutes, with the first six samples collected for baseline. After 60 minutes, rats were stimulated by 0.5 mA foot shock or acoustic startle that were presented at 1-minute interval for 10 minutes. Footshock was included as a condition to allow comparison with the less noxious auditory stimulus used in the behavioral tests. Following 10 minutes of stimulation, samples were collected for an additional 60 minutes. On completion of the experiment, dialysate samples were immediately capped and stored at −80°C until HPLC (EICOM, ECD-300) analysis.

Dyalysate norepinephrine (NE) was determined by HPLC with electrochemical detection. Mobile phase (pH 6.0, 0.1 M sodium phosphate buffer with 5% HPLC grade methanol, 400 mg/L sodium 1-octanesulfonato, 50 mg/L EDTA) were pumped through a reversed-phase column (SC-5ODS, Eicom, 2.1 diameter × 150 mm) at a flow rate of 0.23 mL/min using an ECD detector (450 mV). All substances were identified and quantified by comparing retention times and peak areas with those of external standards. At the beginning of each day, standard mixtures (4 different concentrations, 3 times each) were injected to check and adjust calibration curves. During the course of autoinjection of dialysate fractions, a standard mixture was injected as every fifth sample to monitor and correct calibration curves.

## 3. Results

**Figure 1:**
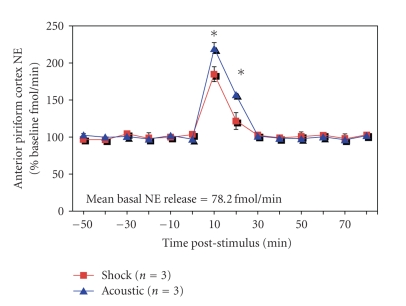
HPLC analysis of microdialysis samples taken from the anterior piriform cortex showed a significant increase in NE content following either siren stimulation as used here, or for comparison, footshock. This increase lasted at least 20 minutes.

We first examined whether an intense nonolfactory sensory experience could elevate norepinephrine levels within the piriform cortex. To test this, we implanted guide cannulas into the anterior piriform cortex to allow microdialysis-HPLC analysis of norepinephrine release in awake rats exposed to an auditory stimulus or to 0.5 mA footshock. Samples were taken every 10 minutes. As shown in Figure 1, mean basal norepinephrine release was stable over approximately 1 hour until the animal was stimulated with either a moderate (0.5 mA) footshock (*n* = 3) or the acoustic stimulus (*n* = 3). With either stimulus, norepinephrine content was significantly elevated 2-fold and stayed elevated for 20 minutes (repeated measures ANOVA, main effect of time: shock, F(13,26) = 50.41, *P* < 0.001; acoustic, F(13,26) = 202.42, *P* < 0.001). Post hoc Fisher tests revealed samples at 10 and 20 minutes poststimulation were significantly more different than all other points (*P* < 0.01).

**Figure 2:**
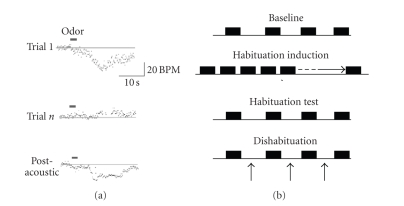
(a) Representative raw recordings of odor-evoked heart rate orienting responses (instantaneous beats/min) in response to odor stimulation before and after habituation, and after presentation of a 108 dB siren. (b) Schematic of experimental design. Odor test pulses (3-4 repeats) were presented with at least 60-second interstimulus intervals to determine mean baseline response magnitude. Then repeated stimuli (20-second ISI) were presented to induce habituation which was tested with 4 spaced stimuli similar to that during baseline (60-second ISI). To induce dishabituation, a 1–2-second siren (upward arrows) was played 10–30 seconds prior to spaced odor stimuli.

**Figure 3:**
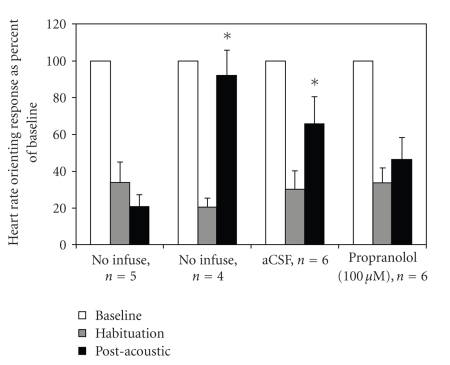
Auditory stimulation induces dishabituation of odor-evoked autonomic reflexes. All groups showed significant habituation of the heart rate orienting reflex (HROR). Initial mean raw response magnitude did not differ between groups. Experimental animals were then presented with the siren and odor responses re-tested. Odor-evoked responses in non-infused and aCSF-infused experimental animals showed a significant increase in postacoustic response magnitude compared to habituation levels (asterisk). Control animals, which received no siren stimulus, and propranolol-infused rats that did receive the acoustic stimulus showed no dishabituation. There was no significant change in basal heart rate across time points in any group.

Given that activation of noradrenergic ß-receptors has been shown to block the synaptic depression that mediates short-term odor habituation [13], we hypothesized that the same auditory stimulus that elevates norepinephrine in the piriform cortex may induce odor dishabituation. The experimental design is described in the Methods and shown in Figure 2. Four groups of rats (noninfused: experimental, *n* = 4, control, *n* = 5; infused: artificial cerebrospinal fluid (aCSF), *n* = 6, propranolol (100 *μ*M), *n* = 6) were tested for odor-evoked bradycardia reflexes. There was no significant difference in initial response magnitude between groups (experimental 22 ± 4.5 beats per min [BPM] decrease; control 18.4 ± 3.5 BPM, aCSF, mean = 18.4 ± 3.5 BPM; propranolol, mean = 22.0 ± 4.5 BPM; ANOVA, N.S.); thus responses are presented as percent of baseline. All groups showed strong habituation to repeated stimulation at 20-second interstimulus intervals (Figure 3). The experimental animals (noninfused, aCSF, and propranolol infused) were then exposed to a loud auditory stimulus, and odor stimulation resumed. Noninfused control animals received no auditory stimulus. Both the noninfused and the aCSF infused experimental animals showed a significant increase to near baseline levels in odor-evoked bradycardia response compared to immediately after habituation training. Noninfused control animals which had no auditory stimulation and acoustically stimulated animals whose piriform cortex was bilaterally infused with the ß-receptor antagonist propranolol showed no significant change from habituation levels (Figure 3). A 2 (time: habituated response, postacoustic response) × 4 (group) repeated measures ANOVA on response magnitude across groups showed a significant time X group interaction (F(3,17) = 6.06, *P* < 0.01). Post hoc Fisher tests revealed that the postacoustic response magnitude in the noninfused experimental animals and the aCSF infused animals was significantly different (*P* < 0.05) from the habituated response magnitude. No significant dishabituation was observed in the noninfused control or propranolol infused rats.

Importantly, there was no significant change in basal heart rate (immediately prior to odor stimulation) between groups across the different time points (ANOVA, time X condition interaction, N.S.). Thus, a sound-induced change in basal heart rate cannot account for the increased bradycardia response. Furthermore, there was no difference in initial basal heart rate between experimental and control conditions.

**Figure 4:**
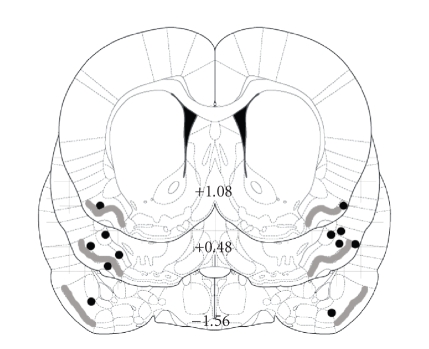
Reconstruction of experiment 3 cannula tip locations in infused rats (black dots) on stereotaxic plates from [20]. All tips were within the anterior piriform cortex, ranging from 1.0 anterior to 1.60 mm posterior to Bregma. Gray line represents layer II of the piriform cortex.

Histological analysis confirmed bilateral cannula tip placements within the anterior piriform cortex (Figure 4).

## 4. Discussion

To our knowledge, this is the first demonstration of dishabituation in mammalian olfaction. The term dishabituation is often used incorrectly to describe responses to test stimuli that differ from the habituation stimulus (e.g., habituate to odor A, test response to odor B). However, dishabituation [1] refers to a recovery of habituation to single stimulus following some other events (e.g., habituate to odor A, stimulus B occurs, and response to odor A is no longer habituated). The present results demonstrate that an auditory stimulus capable of elevating norepinephrine levels in the anterior piriform cortex can produce dishabituation of response to odor. Blockade of noradrenergic ß-receptors within the anterior piriform cortex disrupts this dishabituation. Given previous work demonstrating the necessary and sufficient role of piriform cortical mGluR group III receptors in short-term odor habituation [14–16] and the interaction between noradrenergic ß-receptors and mGluR group III receptor function [13, 17], these results imply that dishabituation of odor-evoked reflexes may be mediated by a reversal of synaptic mechanisms underlying habituation.

Historically, there is a debate over whether dishabituation reflects a reversal of habituation mechanisms, or rather reflects an additional process (e.g., sensitization) superimposed upon habituation. Recent work suggests that both processes may be involved in *Aplysia* [5]. Similarly, although in vitro synaptic physiology [13] and the present results are consistent with reversal of cortical synaptic adaptation contributing to dishabituation of odor responses, it is probable that some sensitization also occurs. For example, activation of the locus coeruleus enhances olfactory bulb mitral cell responsiveness to olfactory nerve input [21] and increases respiratory entrainment of piriform cortical single-unit activity [22], either of which could enhance odor-evoked reflexive responses. Therefore, although noradrenergic ß-receptor blockade within the anterior piriform cortex prevented significant dishabituation here, other, noncortical mechanisms may also contribute to the overall response recovery. These same mechanisms may also contribute to heighten olfactory system responses to novel or important odors given that the locus coeruleus responds most strongly to novel stimuli, showing rapid adaptation with stimulus repetition [23].

Finally, habituation and sensory gating are critical for normal perception, cognition, and emotion, and disruptions are associated with disorders such as schizophrenia and autism spectrum disorder [3, 4]. Together with previous work, the present findings suggest that at least in mammalian olfaction, cortical circuits play a crucial role in these very basic memory phenomena. Understanding how information flow through cortical circuits is modulated during habituation and dishabituation will have important implications for understanding these disorders.

## References

[1] Thompson RF, Spencer WA (1966). Habituation: a model phenomenon for the study of neuronal substrates of behavior. *Psychological Review*.

[2] Frankland PW, Wang Y, Rosner B (2004). Sensorimotor gating abnormalities in young males with fragile X syndrome 
and *Fmr1*-knockout mice. *Molecular Psychiatry*.

[3] Ludewig K, Geyer MA, Vollenweider FX (2003). Deficits in prepulse inhibition and habituation in never-medicated, first-episode schizophrenia. *Biological Psychiatry*.

[4] Perry W, Minassian A, Lopez B, Maron L, Lincoln A (2007). Sensorimotor gating deficits in adults with autism. *Biological Psychiatry*.

[5] Hawkins RD, Cohen TE, Kandel ER (2006). Dishabituation in *Aplysia* can involve either reversal of habituation or superimposed sensitization. *Learning & Memory*.

[6] Kupfermann I, Castellucci V, Pinsker H, Kandel E (1970). Neuronal correlates of habituation and dishabituation of the gill-withdrawal reflex in *Aplysia*. *Science*.

[7] Byrne JH (1982). Analysis of synaptic depression contributing to habituation of gill-withdrawal reflex in *Aplysia* californica. *Journal of Neurophysiology*.

[8] Kandel ER, Brunelli M, Byrne J, Castellucci V (1976). A common presynaptic locus for the synaptic changes underlying short-term habituation and sensitization of the gill-withdrawal reflex in *Aplysia*. *Cold Spring Harbor Symposia on Quantitative Biology*.

[9] Glanzman DL, Mackey SL, Hawkins RD, Dyke AM, Lloyd PE, Kandel ER (1989). Depletion of serotonin in the nervous system of *Aplysia* reduces the behavioral 
enhancement of gill withdrawal as well as the heterosynaptic facilitation produced by tail shock. *The Journal of Neuroscience*.

[10] Kandel ER, Schwartz JH (1982). Molecular biology of learning: modulation of transmitter release. *Science*.

[11] Zhao Y, Klein M (2002). Modulation of the readily releasable pool of transmitter and of excitation-secretion coupling by 
activity and by serotonin at *Aplysia* sensorimotor synapses in culture. *The Journal of Neuroscience*.

[12] Wilson DA (1998). Synaptic correlates of odor habituation in the rat anterior piriform cortex. *Journal of Neurophysiology*.

[13] Best AR, Wilson DA (2004). Coordinate synaptic mechanisms contributing to olfactory cortical adaptation. *The Journal of Neuroscience*.

[14] Best AR, Thompson JV, Fletcher ML, Wilson DA (2005). Cortical metabotropic glutamate receptors contribute to habituation of a simple odor-evoked behavior. *The Journal of Neuroscience*.

[15] McNamara AM, Magidson PD, Linster C, Wilson DA, Cleland TA (2008). Distinct neural mechanisms mediate olfactory memory formation at different timescales. *Learning and Memory*.

[16] Yadon CA, Wilson DA (2005). The role of metabotropic glutamate receptors and cortical adaptation in habituation 
of odor-guided behavior. *Learning and Memory*.

[17] Cai Z, Saugstad JA, Sorensen SD (2001). Cyclic AMP-dependent protein kinase phosphorylates group III metabotropic 
glutamate receptors and inhibits their function as presynaptic receptors. *Journal of Neurochemistry*.

[18] Shipley MT, Ennis M (1996). Functional organization of olfactory system. *Journal of Neurobiology*.

[19] Fletcher M, Wilson DA (2001). Ontogeny of odor discrimination: a method to assess novel odor discrimination in neonatal rats. *Physiology and Behavior*.

[20] Paxinos G, Watson C (2004). *The Rat Brain in Stereotaxic Coordinates*.

[21] Jiang M, Griff ER, Ennis M, Zimmer LA, Shipley MT (1996). Activation of locus coeruleus enhances the responses of olfactory bulb mitral cells to weak olfactory nerve input. *The Journal of Neuroscience*.

[22] Bouret S, Sara SJ (2002). Locus coeruleus activation modulates firing rate and temporal organization of 
odour-induced single-cell responses in rat piriform cortex. *European Journal of Neuroscience*.

[23] Herve-Minvielle A, Sara SJ (1995). Rapid habituation of auditory responses of locus coeruleus cells in anaesthetized and awake rats. *NeuroReport*.

